# Supplementary material for: Higher cost of finance exacerbates a climate investment trap in developing economies
Source: Nat Commun. 2021 Jun 30;12:4046. doi: 10.1038/s41467-021-24305-3 (PMC8245630; doi:10.1038/s41467-021-24305-3)
Supplement: Supplementary file 1 — Supplementary Information [file 41467_2021_24305_MOESM1_ESM.pdf]

Higher cost of finance exacerbates a climate investment trap in developing economies

Supplementary Information

Supplementary Tables

Supplementary Table 1: Data source used for the long-term corporate debt yield

Cost of debt	Geographical reference	Source
Europe	Country level	ECB
US	US	FRED
Japan	Japan	S&P Japan Corporate Bond Index
China	China	S&P China Corporate Bond Index
Mexico	Mexico	S&P Mexico Corporate Bond Index
Emerging Markets	Global emerging markets	FRED
	Emerging Asia	FRED
	Emerging Latin America	FRED

The cost of the debt of the US is also used for Canada and Australia, given the similarities of these markets[1].

Supplementary Table 2: Risk-free rates

	Risk-free rate	
	Germany 10Y Bond yield	US 10Y Treasury Bond yield
Jul 2015 – Jul 2016	0.31%	1.95%

Source: ECB[2], US Department of Treasury[3]

Supplementary Table 3: Solar PV, onshore wind, and offshore wind costs in scenarios (indicative values for Western Europe)

Technology	Cost [$_{2005}$ /kW]		
	2010	2030	2050
Solar PV generation – centralised	2587	633	376
Solar PV generation – decentralised	3118	769	442
Onshore wind generation	1620	969	527
Offshore wind generation	3420	1,749	627

Supplementary Table 4: 16 regions represented in TIAM-UCL and the countries included in each region

Regions and Description	Countries Included
Africa	Algeria, Angola, Benin, Cameroon, Congo, Congo Republic, Egypt, Ethiopia, Gabon, Ghana, Ivory Coast, Kenya, Libya, Morocco, Mozambique, Nigeria, Other Africa, Senegal, South Africa, Sudan, Tanzania, Tunisia, Zambia, Zimbabwe
Australia	Australia and New Zealand
Canada	Canada
Central and South America	Argentina, Bolivia, Brazil, Chile, Colombia, Costa Rica, Cuba, Dominican Republic, Ecuador, El Salvador, Guatemala, Haiti, Honduras, Jamaica, Netherlands Antilles, Nicaragua, Other Latin America, Panama, Paraguay, Peru, Trinidad-Tobago, Uruguay, Venezuela
China	China
Eastern Europe	Albania, Bosnia-Herzegovina, Bulgaria, Croatia, Czech Republic, Hungary, Macedonia, Poland, Romania, Slovakia, Slovenia, Yugoslavia
Former Soviet Union	Armenia, Azerbaijan, Belarus, Estonia, Georgia, Kazakhstan, Kyrgyzstan, Latvia, Lithuania, Moldova, Russia, Tajikistan, Turkmenistan, Ukraine, Uzbekistan
India	India
Japan	Japan
Mexico	Mexico
Middle-East	Bahrain, Cyprus, Iran, Iraq, Israel, Jordan, Kuwait, Lebanon, Oman, Qatar, Saudi Arabia, Syria, Turkey, United Arab Emirates, Yemen
Other Developing Asia	Bangladesh, Brunei, Chinese Taipei, Indonesia, North Korea, Malaysia, Myanmar, Nepal, Other Asia, Pakistan, Philippines, Singapore, Sri Lanka, Thailand, Vietnam
South Korea	South Korea
United Kingdom	United Kingdom
USA	United States of America
Western Europe	Austria, Belgium, Denmark, Finland, France, Germany, Gibraltar, Greece, Greenland, Iceland, Ireland, Italy, Luxembourg, Malta, Netherlands, Norway, Portugal, Spain, Sweden, Switzerland

Supplementary Table 5: Technologies defined as low-carbon and high-carbon

Low-carbon	High-carbon
Renewable electricity generation (hydropower, solar, wind, geothermal and tidal)	Fossil fuel generation without CCS
Biomass conversion (electricity generation and gasification)	Nuclear power
Fossil fuel energy with CCS	
Energy storage	
Carbon sequestration	

Nuclear power has a specific global WACC in TIAM-UCL fixed at 10% that has been kept due to the specific development of this technology.

Supplementary Table 6: Regional power generation in green electricity (green shaded) and total electricity (blue shaded) for the TIAM-UCL regions and the analysed scenarios (in PJ).

Type	*Region*	*Scenario*	2020	2030	2040	2050	2060	2070	2080	2090	2100
Green	AFR	*GBL*	571	1,580	3,071	7,297	13,729	24,050	36,277	44,238	48,168
Green	AFR	*REG*	571	2,116	5,691	11,118	20,021	30,114	40,005	49,343	51,836
Green	AFR	*FAST*	571	1,526	4,096	10,442	19,862	30,172	40,091	49,333	51,605
Green	AFR	*SLOW*	571	1,580	3,618	7,771	16,012	28,548	39,257	48,384	52,408
Green	AUS	*GBL*	298	932	1,362	3,063	3,906	4,855	6,837	9,598	12,159
Green	AUS	*REG*	298	1,007	1,439	3,172	3,930	4,824	6,696	8,157	13,154
Green	AUS	*FAST*	298	959	1,384	3,190	3,841	4,814	6,549	8,249	13,262
Green	AUS	*SLOW*	298	930	1,362	3,063	3,906	4,835	6,623	9,094	12,683
Green	CAN	*GBL*	1,501	1,996	2,672	3,784	7,152	8,545	8,858	8,998	9,818
Green	CAN	*REG*	1,501	2,008	2,659	3,807	7,306	8,556	8,854	9,028	9,812
Green	CAN	*FAST*	1,501	1,990	2,634	3,828	7,321	8,548	8,873	9,027	9,811
Green	CAN	*SLOW*	1,501	1,996	2,669	3,788	7,273	8,542	8,844	8,993	9,813
Green	CHI	*GBL*	5,764	19,792	35,717	71,111	103,732	109,616	111,296	112,310	110,376
Green	CHI	*REG*	5,764	19,942	37,404	74,819	102,095	111,180	112,534	112,634	110,870
Green	CHI	*FAST*	5,764	19,931	37,226	85,050	105,499	111,187	112,569	112,271	111,205
Green	CHI	*SLOW*	5,764	19,786	35,724	71,065	103,352	109,607	112,509	112,353	110,074
Green	CSA	*GBL*	3,163	4,945	7,533	13,347	24,133	33,151	37,791	40,843	46,826
Green	CSA	*FAST*	3,163	4,956	7,550	14,923	25,589	34,683	39,000	43,083	48,749
Green	CSA	*SLOW*	3,163	4,956	7,596	13,700	25,100	34,003	38,896	43,125	48,771
Green	EEU	*GBL*	442	870	1,690	2,474	3,458	3,991	4,397	4,898	5,182
Green	EEU	*REG*	442	870	1,708	2,464	3,447	3,989	4,427	5,007	5,349
Green	EEU	*FAST*	442	871	1,714	2,492	3,514	4,016	4,424	4,968	5,339
Green	EEU	*SLOW*	442	870	1,687	2,464	3,435	3,985	4,408	4,985	5,318
Green	FSU	*GBL*	1,049	3,027	5,777	10,754	15,102	17,647	23,282	27,265	28,831
Green	FSU	*REG*	1,049	3,089	6,157	10,898	15,018	17,701	23,160	27,246	29,147
Green	FSU	*FAST*	1,049	3,031	5,969	10,882	14,663	17,750	23,224	27,332	29,265
Green	FSU	*SLOW*	1,049	3,021	5,803	10,706	15,048	17,643	23,430	27,270	28,990
Green	IND	*GBL*	1,963	5,155	11,128	28,694	39,550	50,694	60,065	68,866	77,308
Green	IND	*REG*	1,963	6,078	12,151	29,159	39,727	51,983	62,111	69,399	77,189
Green	IND	*FAST*	1,963	5,339	11,825	29,133	40,009	52,187	62,002	69,200	77,204
Green	IND	*SLOW*	1,963	5,207	11,192	28,855	39,679	51,126	60,762	69,312	77,321
Green	JPN	*GBL*	785	1,977	2,465	4,100	6,844	8,022	8,388	8,442	8,645
Green	JPN	*REG*	785	1,985	2,469	4,079	6,886	8,137	8,133	8,383	8,609
Green	JPN	*FAST*	785	1,976	2,460	4,107	6,876	8,141	8,167	8,420	8,625
Green	JPN	*SLOW*	785	1,976	2,462	4,097	6,901	8,166	8,342	8,574	8,753
Green	MEA	*GBL*	501	1,234	5,074	10,205	16,697	27,628	31,385	31,277	34,458
Green	MEA	*REG*	501	1,344	5,371	11,121	19,143	28,396	31,462	31,563	35,647
Green	MEA	*FAST*	501	1,337	5,380	11,321	19,058	28,262	31,466	31,572	35,648
Green	MEA	*SLOW*	501	1,337	5,370	11,131	19,143	28,101	31,466	31,537	35,644
Green	MEX	*GBL*	183	460	1,040	1,935	3,526	4,564	5,322	5,943	6,519
Green	MEX	*REG*	183	501	1,219	2,232	4,230	5,060	5,672	6,356	6,846
Green	MEX	*FAST*	183	429	1,079	2,351	4,252	5,072	5,709	6,363	6,846
Green	MEX	*SLOW*	183	464	1,051	1,976	3,744	4,907	5,665	6,336	6,846
Green	ODA	*GBL*	1,217	2,892	8,661	17,496	30,632	46,983	58,089	64,255	69,341
Green	ODA	*REG*	1,217	2,921	8,938	17,480	31,256	48,291	59,570	66,334	69,672
Green	ODA	*FAST*	1,217	2,827	8,727	17,865	31,292	48,329	59,618	66,712	69,639
Green	ODA	*SLOW*	1,217	2,827	8,656	17,735	31,297	48,010	58,917	65,557	69,426
Green	SKO	*GBL*	88	350	747	1,399	1,767	2,524	3,195	3,714	3,683
Green	SKO	*REG*	88	355	756	1,521	2,075	3,082	3,779	4,184	4,129
Green	SKO	*FAST*	88	343	748	1,597	2,098	3,098	3,788	4,203	4,149
Green	SKO	*SLOW*	88	350	748	1,498	1,930	2,727	3,447	3,959	4,084
Green	UK	*GBL*	327	830	1,731	4,152	6,267	7,154	7,745	8,114	8,714
Green	UK	*REG*	327	812	1,741	4,152	6,280	7,166	7,732	8,169	8,812
Green	UK	*FAST*	327	827	1,773	4,274	6,334	7,163	7,730	8,165	8,807
Green	UK	*SLOW*	327	827	1,733	4,154	6,272	7,160	7,737	8,147	8,788
Green	USA	*GBL*	2,691	8,443	17,666	34,641	53,869	60,806	63,840	65,405	70,017
Green	USA	*REG*	2,691	8,429	17,741	34,896	53,712	60,909	63,867	66,590	70,155
Green	USA	*FAST*	2,691	8,426	17,791	35,536	53,798	61,348	63,990	66,816	70,252
Green	USA	*SLOW*	2,691	8,440	17,708	34,800	53,889	60,901	63,869	66,007	70,448
Green	WEU	*GBL*	3,799	6,165	8,529	15,466	20,667	21,588	21,278	22,202	23,068
Green	WEU	*REG*	3,799	6,125	8,550	15,355	20,404	21,317	21,208	21,486	22,536
Green	WEU	*FAST*	3,799	6,155	8,665	15,794	20,356	21,061	21,125	21,467	22,627

Green	WEU	*SLOW*	3,799	6,159	8,580	15,400	20,291	21,266	21,214	21,839	22,610
Total	AFR	*GBL*	2,911	3,266	4,009	7,682	13,830	24,107	36,295	44,240	48,274
Total	AFR	*REG*	2,911	3,338	6,213	11,251	20,137	30,189	40,023	49,345	51,836
Total	AFR	*FAST*	2,911	3,262	5,634	10,610	19,977	30,194	40,095	49,335	51,605
Total	AFR	*SLOW*	2,911	3,294	4,661	8,430	16,138	28,616	39,274	48,386	52,408
Total	AUS	*GBL*	1,180	1,409	1,568	3,093	3,920	4,855	6,837	9,598	12,159
Total	AUS	*REG*	1,180	1,400	1,575	3,198	3,945	4,824	6,696	8,157	13,154
Total	AUS	*FAST*	1,180	1,391	1,550	3,213	3,854	4,814	6,549	8,249	13,262
Total	AUS	*SLOW*	1,180	1,403	1,568	3,093	3,921	4,835	6,623	9,094	12,683
Total	CAN	*GBL*	2,491	2,815	2,898	3,921	7,218	8,595	8,891	9,014	9,818
Total	CAN	*REG*	2,491	2,823	2,883	3,943	7,372	8,606	8,887	9,044	9,812
Total	CAN	*FAST*	2,491	2,821	2,888	3,966	7,387	8,598	8,906	9,044	9,811
Total	CAN	*SLOW*	2,491	2,814	2,895	3,925	7,339	8,592	8,877	9,009	9,813
Total	CHI	*GBL*	26,849	33,058	44,314	73,501	105,225	111,493	114,006	115,947	115,198
Total	CHI	*REG*	26,849	33,549	46,606	77,417	103,405	113,057	114,758	115,673	115,238
Total	CHI	*FAST*	26,849	33,218	46,052	87,330	106,419	112,992	114,606	115,244	115,492
Total	CHI	*SLOW*	26,849	33,050	44,424	73,469	104,592	111,371	114,671	115,370	114,415
Total	CSA	*GBL*	4,015	5,046	7,626	13,492	24,271	33,245	37,814	40,846	46,826
Total	CSA	*REG*	4,015	5,139	7,824	14,846	25,550	34,476	38,993	43,084	48,774
Total	CSA	*FAST*	4,015	5,057	7,609	14,978	25,636	34,722	39,006	43,086	48,749
Total	CSA	*SLOW*	4,015	5,057	7,693	13,853	25,245	34,100	38,920	43,128	48,771
Total	EEU	*GBL*	2,004	2,215	2,483	3,333	3,986	4,809	5,581	6,366	6,966
Total	EEU	*REG*	2,004	2,216	2,481	3,302	3,958	4,781	5,570	6,439	7,094
Total	EEU	*FAST*	2,004	2,216	2,488	3,333	3,988	4,779	5,557	6,399	7,083
Total	EEU	*SLOW*	2,004	2,215	2,480	3,320	3,976	4,815	5,593	6,450	7,098
Total	FSU	*GBL*	5,477	8,661	10,036	12,781	16,568	18,330	23,588	27,344	28,866
Total	FSU	*REG*	5,477	8,689	10,059	12,776	16,340	18,209	23,382	27,290	29,147
Total	FSU	*FAST*	5,477	8,665	9,977	12,784	16,008	18,282	23,454	27,376	29,265
Total	FSU	*SLOW*	5,477	8,657	10,031	12,784	16,566	18,335	23,694	27,313	28,990
Total	IND	*GBL*	5,499	6,498	12,179	30,172	40,838	51,708	60,679	68,914	77,308
Total	IND	*REG*	5,499	7,179	13,240	30,721	41,074	53,033	62,725	69,447	77,189
Total	IND	*FAST*	5,499	6,580	12,827	30,529	41,225	52,996	62,616	69,248	77,204
Total	IND	*SLOW*	5,499	6,551	12,253	30,365	40,995	52,162	61,376	69,360	77,321
Total	JPN	*GBL*	3,704	4,206	3,925	5,031	7,121	8,213	8,499	8,497	8,645
Total	JPN	*REG*	3,704	4,206	3,967	5,041	7,170	8,329	8,245	8,438	8,609
Total	JPN	*FAST*	3,704	4,204	3,939	5,073	7,138	8,320	8,276	8,474	8,625
Total	JPN	*SLOW*	3,704	4,205	3,927	5,048	7,183	8,361	8,455	8,629	8,753
Total	MEA	*GBL*	3,741	5,583	7,271	13,457	19,458	27,854	31,461	31,277	34,458
Total	MEA	*REG*	3,741	5,580	7,356	13,712	20,188	28,503	31,478	31,563	35,647
Total	MEA	*FAST*	3,741	5,580	7,389	13,943	20,018	28,343	31,477	31,572	35,648
Total	MEA	*SLOW*	3,741	5,580	7,375	13,735	20,187	28,207	31,479	31,537	35,644
Total	MEX	*GBL*	802	863	1,127	1,948	3,534	4,570	5,326	5,945	6,519
Total	MEX	*REG*	802	908	1,268	2,245	4,238	5,066	5,676	6,358	6,846
Total	MEX	*FAST*	802	879	1,185	2,364	4,260	5,077	5,713	6,365	6,846
Total	MEX	*SLOW*	802	876	1,155	1,989	3,752	4,913	5,669	6,338	6,846
Total	ODA	*GBL*	4,590	5,735	9,550	18,604	30,946	47,166	58,159	64,263	69,341
Total	ODA	*REG*	4,590	5,702	9,668	18,437	31,568	48,481	59,646	66,342	69,672
Total	ODA	*FAST*	4,590	5,723	9,703	18,712	31,591	48,496	59,700	66,720	69,639
Total	ODA	*SLOW*	4,590	5,727	9,562	18,649	31,607	48,186	59,002	65,565	69,426
Total	SKO	*GBL*	1,975	2,099	2,389	3,192	3,835	4,948	5,736	6,448	6,613
Total	SKO	*REG*	1,975	2,099	2,390	3,229	3,903	5,125	5,860	6,543	6,714
Total	SKO	*FAST*	1,975	2,100	2,389	3,325	3,915	5,132	5,860	6,542	6,720
Total	SKO	*SLOW*	1,975	2,099	2,389	3,220	3,857	4,981	5,792	6,505	6,709
Total	UK	*GBL*	1,350	1,541	1,870	4,219	6,304	7,154	7,745	8,114	8,714
Total	UK	*REG*	1,350	1,541	1,881	4,220	6,318	7,166	7,732	8,169	8,812
Total	UK	*FAST*	1,350	1,541	1,912	4,341	6,371	7,163	7,730	8,165	8,807
Total	UK	*SLOW*	1,350	1,541	1,872	4,222	6,309	7,160	7,737	8,147	8,788
Total	USA	*GBL*	16,084	17,311	21,078	36,411	54,764	61,243	64,132	65,551	70,017
Total	USA	*REG*	16,084	17,310	21,086	36,659	54,601	61,348	64,159	66,735	70,155
Total	USA	*FAST*	16,084	17,334	21,048	37,275	54,665	61,785	64,281	66,962	70,252
Total	USA	*SLOW*	16,084	17,317	21,079	36,564	54,781	61,339	64,160	66,153	70,448
Total	WEU	*GBL*	9,597	10,366	9,978	15,639	20,768	21,610	21,278	22,202	23,068
Total	WEU	*REG*	9,597	10,334	9,980	15,529	20,506	21,341	21,208	21,486	22,536
Total	WEU	*FAST*	9,597	10,305	9,951	15,914	20,406	21,065	21,125	21,467	22,627
Total	WEU	*SLOW*	9,597	10,333	9,976	15,571	20,391	21,291	21,214	21,839	22,610

Supplementary Table 7: Regional investment levels in green electricity generation (green shaded) and total electricity generation (blue shaded) for the TIAM-UCL regions and the analysed scenarios (in M$2005).

Type	*Region*	*Scenario*	2020	2030	2040	2050	2060	2070	2080	2090	2100
Green	*AFR*	*GBL*	571	1,580	3,071	7,297	13,729	24,050	36,277	44,238	48,168
Green	*AFR*	*REG*	571	2,116	5,691	11,118	20,021	30,114	40,005	49,343	51,836
Green	*AFR*	*FAST*	571	1,526	4,096	10,442	19,862	30,172	40,091	49,333	51,605
Green	*AFR*	*SLOW*	571	1,580	3,618	7,771	16,012	28,548	39,257	48,384	52,408
Green	*AUS*	*GBL*	298	932	1,362	3,063	3,906	4,855	6,837	9,598	12,159
Green	*AUS*	*REG*	298	1,007	1,439	3,172	3,930	4,824	6,696	8,157	13,154
Green	*AUS*	*FAST*	298	959	1,384	3,190	3,841	4,814	6,549	8,249	13,262
Green	*AUS*	*SLOW*	298	930	1,362	3,063	3,906	4,835	6,623	9,094	12,683
Green	*CAN*	*GBL*	1,501	1,996	2,672	3,784	7,152	8,545	8,858	8,998	9,818
Green	*CAN*	*REG*	1,501	2,008	2,659	3,807	7,306	8,556	8,854	9,028	9,812
Green	*CAN*	*FAST*	1,501	1,990	2,634	3,828	7,321	8,548	8,873	9,027	9,811
Green	*CAN*	*SLOW*	1,501	1,996	2,669	3,788	7,273	8,542	8,844	8,993	9,813
Green	*CHI*	*GBL*	5,764	19,792	35,717	71,111	103,732	109,616	111,296	112,310	110,376
Green	*CHI*	*REG*	5,764	19,942	37,404	74,819	102,095	111,180	112,534	112,634	110,870
Green	*CHI*	*FAST*	5,764	19,931	37,226	85,050	105,499	111,187	112,569	112,271	111,205
Green	*CHI*	*SLOW*	5,764	19,786	35,724	71,065	103,352	109,607	112,509	112,353	110,074
Green	*CSA*	*GBL*	3,163	4,945	7,533	13,347	24,133	33,151	37,791	40,843	46,826
Green	*CSA*	*REG*	3,163	5,038	7,720	14,683	25,395	34,376	38,968	43,081	48,774
Green	*CSA*	*FAST*	3,163	4,956	7,550	14,923	25,589	34,683	39,000	43,083	48,749
Green	*CSA*	*SLOW*	3,163	4,956	7,596	13,700	25,100	34,003	38,896	43,125	48,771
Green	*EEU*	*GBL*	442	870	1,690	2,474	3,458	3,991	4,397	4,898	5,182
Green	*EEU*	*REG*	442	870	1,708	2,464	3,447	3,989	4,427	5,007	5,349
Green	*EEU*	*FAST*	442	871	1,714	2,492	3,514	4,016	4,424	4,968	5,339
Green	*EEU*	*SLOW*	442	870	1,687	2,464	3,435	3,985	4,408	4,985	5,318
Green	*FSU*	*GBL*	1,049	3,027	5,777	10,754	15,102	17,647	23,282	27,265	28,831
Green	*FSU*	*REG*	1,049	3,089	6,157	10,898	15,018	17,701	23,160	27,246	29,147
Green	*FSU*	*FAST*	1,049	3,031	5,969	10,882	14,663	17,750	23,224	27,332	29,265
Green	*FSU*	*SLOW*	1,049	3,021	5,803	10,706	15,048	17,643	23,430	27,270	28,990
Green	*IND*	*GBL*	1,963	5,155	11,128	28,694	39,550	50,694	60,065	68,866	77,308
Green	*IND*	*REG*	1,963	6,078	12,151	29,159	39,727	51,983	62,111	69,399	77,189
Green	*IND*	*FAST*	1,963	5,339	11,825	29,133	40,009	52,187	62,002	69,200	77,204
Green	*IND*	*SLOW*	1,963	5,207	11,192	28,855	39,679	51,126	60,762	69,312	77,321
Green	*JPN*	*GBL*	785	1,977	2,465	4,100	6,844	8,022	8,388	8,442	8,645
Green	*JPN*	*REG*	785	1,985	2,469	4,079	6,886	8,137	8,133	8,383	8,609
Green	*JPN*	*FAST*	785	1,976	2,460	4,107	6,876	8,141	8,167	8,420	8,625
Green	*JPN*	*SLOW*	785	1,976	2,462	4,097	6,901	8,166	8,342	8,574	8,753
Green	*MEA*	*GBL*	501	1,234	5,074	10,205	16,697	27,628	31,385	31,277	34,458
Green	*MEA*	*REG*	501	1,344	5,371	11,121	19,143	28,396	31,462	31,563	35,647
Green	*MEA*	*FAST*	501	1,337	5,380	11,321	19,058	28,262	31,466	31,572	35,648
Green	*MEA*	*SLOW*	501	1,337	5,370	11,131	19,143	28,101	31,466	31,537	35,644
Green	*MEX*	*GBL*	183	460	1,040	1,935	3,526	4,564	5,322	5,943	6,519
Green	*MEX*	*REG*	183	501	1,219	2,232	4,230	5,060	5,672	6,356	6,846
Green	*MEX*	*FAST*	183	429	1,079	2,351	4,252	5,072	5,709	6,363	6,846
Green	*MEX*	*SLOW*	183	464	1,051	1,976	3,744	4,907	5,665	6,336	6,846
Green	*ODA*	*GBL*	1,217	2,892	8,661	17,496	30,632	46,983	58,089	64,255	69,341
Green	*ODA*	*REG*	1,217	2,921	8,938	17,480	31,256	48,291	59,570	66,334	69,672
Green	*ODA*	*FAST*	1,217	2,827	8,727	17,865	31,292	48,329	59,618	66,712	69,639
Green	*ODA*	*SLOW*	1,217	2,827	8,656	17,735	31,297	48,010	58,917	65,557	69,426
Green	*SKO*	*GBL*	88	350	747	1,399	1,767	2,524	3,195	3,714	3,683
Green	*SKO*	*REG*	88	355	756	1,521	2,075	3,082	3,779	4,184	4,129
Green	*SKO*	*FAST*	88	343	748	1,597	2,098	3,098	3,788	4,203	4,149
Green	*SKO*	*SLOW*	88	350	748	1,498	1,930	2,727	3,447	3,959	4,084
Green	*UK*	*GBL*	327	830	1,731	4,152	6,267	7,154	7,745	8,114	8,714
Green	*UK*	*REG*	327	812	1,741	4,152	6,280	7,166	7,732	8,169	8,812
Green	*UK*	*FAST*	327	827	1,773	4,274	6,334	7,163	7,730	8,165	8,807
Green	*UK*	*SLOW*	327	827	1,733	4,154	6,272	7,160	7,737	8,147	8,788
Green	*USA*	*GBL*	2,691	8,443	17,666	34,641	53,869	60,806	63,840	65,405	70,017
Green	*USA*	*REG*	2,691	8,429	17,741	34,896	53,712	60,909	63,867	66,590	70,155
Green	*USA*	*FAST*	2,691	8,426	17,791	35,536	53,798	61,348	63,990	66,816	70,252
Green	*USA*	*SLOW*	2,691	8,440	17,708	34,800	53,889	60,901	63,869	66,007	70,448
Green	*WEU*	*GBL*	3,799	6,165	8,529	15,466	20,667	21,588	21,278	22,202	23,068
Green	*WEU*	*REG*	3,799	6,125	8,550	15,355	20,404	21,317	21,208	21,486	22,536

Green	*WEU*	*FAST*	3,799	6,155	8,665	15,794	20,356	21,061	21,125	21,467	22,627
Green	*WEU*	*SLOW*	3,799	6,159	8,580	15,400	20,291	21,266	21,214	21,839	22,610
Total	*AFR*	*GBL*	4,643	4,693	5,644	9,347	15,789	26,120	38,357	46,328	50,268
Total	*AFR*	*REG*	2,911	3,802	6,629	11,503	20,122	30,171	40,024	49,345	51,943
Total	*AFR*	*FAST*	2,911	2,748	4,618	10,576	19,977	30,247	40,110	49,335	51,605
Total	*AFR*	*SLOW*	2,911	3,316	5,156	7,938	16,128	28,570	39,261	48,386	52,408
Total	*AUS*	*GBL*	2,639	2,535	2,645	4,893	5,674	6,199	7,849	9,864	13,105
Total	*AUS*	*REG*	1,180	1,484	1,645	3,202	3,945	4,824	6,696	8,157	13,154
Total	*AUS*	*FAST*	1,180	1,352	1,520	3,215	3,856	4,814	6,549	8,249	13,262
Total	*AUS*	*SLOW*	1,180	1,363	1,528	3,087	3,919	4,835	6,623	9,094	12,683
Total	*CAN*	*GBL*	2,383	2,626	3,152	4,013	7,234	8,630	8,939	9,081	9,878
Total	*CAN*	*REG*	2,491	2,826	2,885	3,943	7,372	8,606	8,887	9,044	9,812
Total	*CAN*	*FAST*	2,491	2,806	2,857	3,965	7,387	8,598	8,906	9,044	9,811
Total	*CAN*	*SLOW*	2,491	2,827	2,922	3,925	7,339	8,592	8,877	9,009	9,813
Total	*CHI*	*GBL*	6,755	20,732	36,406	71,971	103,857	109,707	111,338	112,343	110,393
Total	*CHI*	*REG*	26,849	33,208	46,000	77,209	103,589	113,057	115,245	116,271	115,692
Total	*CHI*	*FAST*	26,849	33,539	46,427	87,648	106,809	113,064	114,792	115,309	115,573
Total	*CHI*	*SLOW*	26,849	33,073	44,551	73,345	104,271	111,412	114,546	115,327	114,361
Total	*CSA*	*GBL*	24,248	22,434	25,736	22,565	27,784	34,264	38,966	42,273	48,845
Total	*CSA*	*REG*	4,015	5,139	7,813	14,829	25,533	34,470	38,992	43,084	48,774
Total	*CSA*	*FAST*	4,015	5,057	7,653	15,085	25,744	34,784	39,025	43,086	48,749
Total	*CSA*	*SLOW*	4,015	5,057	7,655	13,755	25,147	34,042	38,902	43,128	48,771
Total	*EEU*	*GBL*	1,293	1,128	1,793	2,651	3,692	4,251	4,647	5,157	5,369
Total	*EEU*	*REG*	2,004	2,215	2,501	3,323	3,975	4,807	5,611	6,476	7,133
Total	*EEU*	*FAST*	2,004	2,217	2,487	3,330	4,025	4,809	5,567	6,399	7,083
Total	*EEU*	*SLOW*	2,004	2,215	2,461	3,305	3,909	4,748	5,541	6,416	7,062
Total	*FSU*	*GBL*	2,612	4,828	8,032	13,410	17,560	20,104	25,360	29,345	30,928
Total	*FSU*	*REG*	5,477	8,722	10,416	12,924	16,484	18,383	23,466	27,325	29,182
Total	*FSU*	*FAST*	5,477	8,632	9,871	12,760	15,984	18,258	23,445	27,376	29,265
Total	*FSU*	*SLOW*	5,477	8,655	9,811	12,607	16,394	18,176	23,660	27,313	28,990
Total	*IND*	*GBL*	6,390	11,811	19,038	38,316	46,748	56,167	63,555	70,179	78,509
Total	*IND*	*REG*	5,499	7,421	13,202	30,637	41,015	52,997	62,725	69,447	77,189
Total	*IND*	*FAST*	5,499	6,440	12,914	30,695	41,355	53,237	62,616	69,248	77,204
Total	*IND*	*SLOW*	5,499	6,448	12,195	30,251	40,896	51,935	61,376	69,360	77,321
Total	*JPN*	*GBL*	4,321	4,614	6,170	6,288	8,303	9,192	9,043	8,491	8,645
Total	*JPN*	*REG*	3,704	4,214	3,929	5,009	7,164	8,328	8,244	8,438	8,609
Total	*JPN*	*FAST*	3,704	4,197	3,958	5,069	7,160	8,333	8,279	8,474	8,625
Total	*JPN*	*SLOW*	3,704	4,205	3,941	5,063	7,163	8,345	8,452	8,629	8,753
Total	*MEA*	*GBL*	3,420	3,763	7,272	12,196	17,448	28,146	31,795	31,632	34,745
Total	*MEA*	*REG*	3,741	5,693	7,568	14,373	21,903	28,622	31,538	31,563	35,647
Total	*MEA*	*FAST*	3,741	5,574	7,365	13,913	20,102	28,369	31,482	31,572	35,648
Total	*MEA*	*SLOW*	3,741	5,580	7,380	13,753	20,103	28,183	31,477	31,537	35,644
Total	*MEX*	*GBL*	3,424	4,938	3,796	5,658	7,834	10,440	11,545	11,864	11,180
Total	*MEX*	*REG*	802	904	1,306	2,244	4,238	5,066	5,676	6,358	6,846
Total	*MEX*	*FAST*	802	836	1,127	2,364	4,260	5,077	5,713	6,365	6,846
Total	*MEX*	*SLOW*	802	914	1,157	1,989	3,751	4,913	5,669	6,338	6,846
Total	*ODA*	*GBL*	1,835	3,480	9,252	18,045	30,721	47,019	58,099	64,257	69,341
Total	*ODA*	*REG*	4,590	5,763	9,827	18,588	31,571	48,474	59,639	66,342	69,672
Total	*ODA*	*FAST*	4,590	5,608	9,457	18,823	31,604	48,520	59,694	66,720	69,639
Total	*ODA*	*SLOW*	4,590	5,723	9,632	18,582	31,597	48,176	58,999	65,565	69,426
Total	*SKO*	*GBL*	3,462	3,595	2,837	3,560	2,827	3,345	3,654	4,424	4,434
Total	*SKO*	*REG*	1,975	2,104	2,397	3,314	4,143	5,507	6,320	6,918	7,060
Total	*SKO*	*FAST*	1,975	2,086	2,382	3,305	3,926	5,141	5,869	6,562	6,733
Total	*SKO*	*SLOW*	1,975	2,107	2,388	3,225	3,747	4,762	5,519	6,298	6,656
Total	*UK*	*GBL*	2,214	2,728	3,808	6,401	7,982	8,812	9,295	9,524	10,091
Total	*UK*	*REG*	1,350	1,522	1,880	4,220	6,317	7,166	7,732	8,169	8,812
Total	*UK*	*FAST*	1,350	1,556	1,913	4,343	6,372	7,163	7,730	8,165	8,807
Total	*UK*	*SLOW*	1,350	1,540	1,872	4,221	6,309	7,160	7,737	8,147	8,788
Total	*USA*	*GBL*	3,714	9,225	17,913	34,748	53,943	60,834	63,845	65,417	70,029
Total	*USA*	*REG*	16,084	17,297	21,152	36,665	54,607	61,347	64,159	66,735	70,155
Total	*USA*	*FAST*	16,084	17,307	21,136	37,299	54,687	61,787	64,281	66,962	70,252
Total	*USA*	*SLOW*	16,084	17,348	20,965	36,539	54,756	61,339	64,160	66,153	70,448
Total	*WEU*	*GBL*	17,192	15,523	15,139	18,347	21,982	22,317	21,615	22,348	23,068
Total	*WEU*	*REG*	9,597	10,326	9,999	15,527	20,505	21,339	21,208	21,486	22,536
Total	*WEU*	*FAST*	9,597	10,364	10,094	15,968	20,458	21,086	21,125	21,467	22,627
Total	*WEU*	*SLOW*	9,597	10,309	9,866	15,520	20,341	21,270	21,214	21,839	22,610

Supplementary Figures

Supplementary Figure 1: Green electricity generation and investment per year for the two region groups

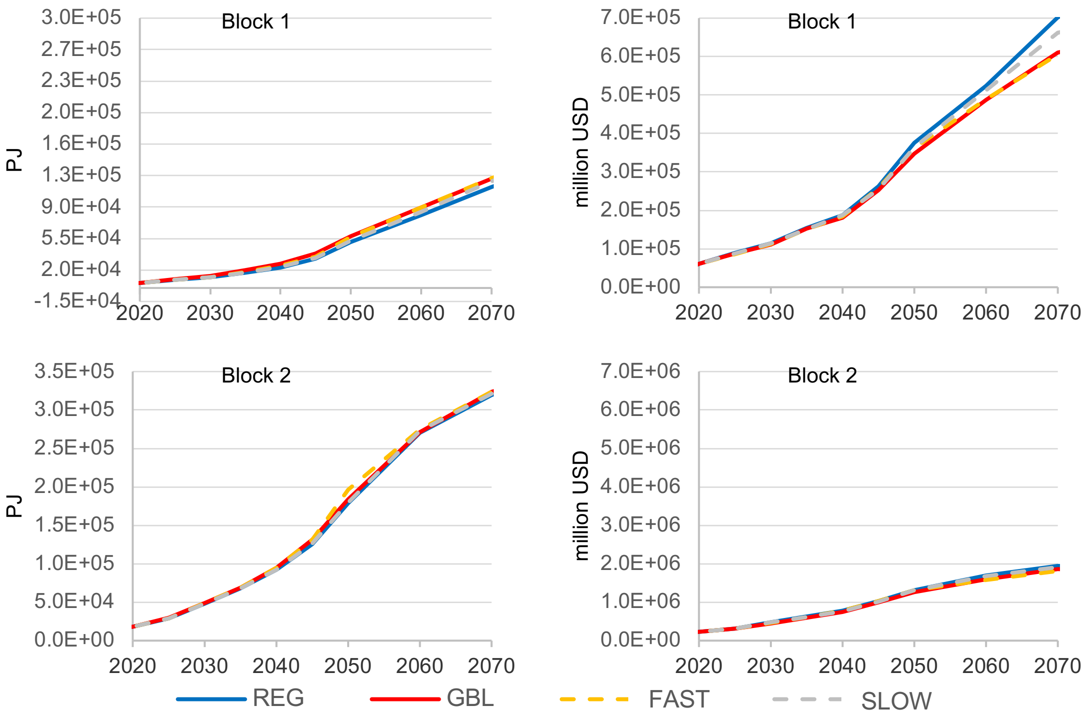

The left hand panels show green electricity generation in PJ, while the right hand panels show investment per year for the two region groups

Supplementary notes

Supplementary note 1

Global results for the different scenarios are available. We focus on power generation and provide the total (including brown and green power) and green electricity production. Supplementary Figure 1 shows the global results in a simplified manner by aggregating the 16 regions of TIAM-UCL in 2 blocks. The first block represents the regions experiencing large change in WACC values between the REG and GBL scenarios (namely: Africa, Central and South America, India and Mexico) and the second represents the remaining regions which experience weak or no changes in their WACCs (respectively referred to Block 1 and Block 2 in the Figure). The results are similar to those discussed in the main text: in the regions with high WACC values (Block 1), lowering the WACC increases the green electricity generation in the 2C pathway, while maintaining a lower level of investment per generated unit. In the same way, a faster WACC reduction (FAST) increases early green generation production at least costs in the Block 1 countries. Finally, the specific electricity generation (total and green) at the regional level (for the 16 regions and the 5 scenarios) are given in supplementary Table 6 and the corresponding investments in supplementary Table 7.

References

1. Miville, M., & Bernier, A. (1999). The Corporate Bond Market in Canada. Bank of Canada Review.
2. ECB (2016). Euro area yield statistics. https://www.ecb.europa.eu/stats/financial_markets_and_interest_rates/euro_area_yield_curves/html/index.en.html
3. US Department of Treasury (2016). Treasury Yield Curve Rates statistics https://www.treasury.gov/resource-center/data-chart-center/interest-rates/Pages/TextView.aspx?data=realyield
