# Supplementary material for: Higher cost of finance exacerbates a climate investment trap in developing economies
Source: Nat Commun. 2021 Jun 30;12:4046. doi: 10.1038/s41467-021-24305-3 (PMC8245630; doi:10.1038/s41467-021-24305-3)
Supplement: Supplementary file 3 — Reporting Summary [file 41467_2021_24305_MOESM3_ESM.pdf]

nature research

Corresponding author(s): Nadia Ameli

Last updated by author(s): April 30, 2021

Reporting Summary

Nature Research wishes to improve the reproducibility of the work that we publish. This form provides structure for consistency and transparency in reporting. For further information on Nature Research policies, see our Editorial Policies and the Editorial Policy Checklist.

Statistics

For all statistical analyses, confirm that the following items are present in the figure legend, table legend, main text, or Methods section.

n/a	Confirmed	
☐	☒	The exact sample size (*n*) for each experimental group/condition, given as a discrete number and unit of measurement
☒	☐	A statement on whether measurements were taken from distinct samples or whether the same sample was measured repeatedly
☒	☐	The statistical test(s) used AND whether they are one- or two-sided<br>*Only common tests should be described solely by name; describe more complex techniques in the Methods section.*
☒	☐	A description of all covariates tested
☒	☐	A description of any assumptions or corrections, such as tests of normality and adjustment for multiple comparisons
☒	☐	A full description of the statistical parameters including central tendency (e.g. means) or other basic estimates (e.g. regression coefficient) AND variation (e.g. standard deviation) or associated estimates of uncertainty (e.g. confidence intervals)
☒	☐	For null hypothesis testing, the test statistic (e.g. *F*, *t*, *r*) with confidence intervals, effect sizes, degrees of freedom and *P* value noted<br>*Give P values as exact values whenever suitable.*
☒	☐	For Bayesian analysis, information on the choice of priors and Markov chain Monte Carlo settings
☒	☐	For hierarchical and complex designs, identification of the appropriate level for tests and full reporting of outcomes
☒	☐	Estimates of effect sizes (e.g. Cohen's *d*, Pearson's *r*), indicating how they were calculated

Our web collection on statistics for biologists contains articles on many of the points above.

Software and code

Policy information about availability of computer code

Data collection	We collected WACC variable data manually from different sources and used excel to derive country/region WACC values
Data analysis	The code underlying the TIAM-UCL model is available at this link:<br>https://doi.org/10.5281/zenodo.3930657

For manuscripts utilizing custom algorithms or software that are central to the research but not yet described in published literature, software must be made available to editors and reviewers. We strongly encourage code deposition in a community repository (e.g. GitHub). See the Nature Research guidelines for submitting code & software for further information.

Data

Policy information about availability of data

All manuscripts must include a data availability statement. This statement should provide the following information, where applicable:

- Accession codes, unique identifiers, or web links for publicly available datasets
- A list of figures that have associated raw data
- A description of any restrictions on data availability

The results data and key source data are provided with this paper. Other datasets used in the determination of the WACC values include the Damodaran dataset (accessed here http://pages.stern.nyu.edu/~adamodar/New_Home_Page/datacurrent.html), the Euro area yield statistics (accessed here https://www.ecb.europa.eu/stats/financial_markets_and_interest_rates/euro_area_yield_curves/html/index.en.html), the US Treasury Yield Curve Rates statistics (accessed here https://www.treasury.gov/resource-center/data-chart-center/interest-rates/Pages/TextView.aspx?data=realyield) and the KPMG Corporate tax rates dataset (accessed here https://home.kpmg/xx/en/home/services/tax/tax-tools-and-resources/tax-rates-online/corporate-tax-rates-table.html).
Other modeling input assumptions are available on reasonable request.

Field-specific reporting

Please select the one below that is the best fit for your research. If you are not sure, read the appropriate sections before making your selection.

☐ Life sciences ☒ Behavioural & social sciences ☐ Ecological, evolutionary & environmental sciences

For a reference copy of the document with all sections, see nature.com/documents/nr-reporting-summary-flat.pdf

Behavioural & social sciences study design

All studies must disclose on these points even when the disclosure is negative.

Study description	This study introduces regionally-differentiated cost of capital values to the TIAM-UCL global energy systems model to assess how these assumptions for the power sector affect patterns of cost-optimal investment for the energy transition consistent with the 2°C global target. The cost of capital values are based on quantitative data collected from different data sources. The TIAM-UCL model is used to perform the analysis. TIAM-UCL is a technology-rich bottom-up cost optimisation model that determines the least-cost energy and technology mix that meets future energy demands while respecting technical, economic and policy constraints, such that societal welfare is maximised.
Research sample	We used a new WACC database covering developed and developing economies. Data variables, their aggregation and sources are reported in the Method section, while more detail is available in Ameli et al (2017). This dataset was selected for its detailed coverage at global level, thus allowing us to analyze how modelled global decarbonisation pathways are impacted by different cost of capital assumptions. In particular, the dataset includes WACC data at country level for most of European countries, the US, China, Australia, Canada, Japan and Mexico. While data at regional level relates mainly to developing economies, including Latin American countries (including Brazil and Andean, a compound index of Chile, Colombia, Peru), Asian countries (emerging Asian economies as whole) and global emerging countries (including all other emerging economies except those in Asia and Latin America).
Sampling strategy	We collected the WACC values using a clustering sampling strategy, where clusters are identified at country or regional level (depending on data availability). The WACC values are based on estimates of more 40,000+ individual companies at industry level in a given country or region, including the cost of financing for upstream and downstream companies along the value chain. Sample size tests were not performed given the large number of companies included in the dataset (Damodaran dataset).
Data collection	Data collection from existing available resources was performed as desk research and data were stored in researchers laptop.
Timing	Data collection refers to values in 2016 - such data were collected from January to March 2017
Data exclusions	No data were excluded from the analysis.
Non-participation	As our analysis is based on public available datasets, we do not have access to this info.
Randomization	Randomization is not relevant to this study as we do not measure any reatment effects on specific groups.

Reporting for specific materials, systems and methods

We require information from authors about some types of materials, experimental systems and methods used in many studies. Here, indicate whether each material, system or method listed is relevant to your study. If you are not sure if a list item applies to your research, read the appropriate section before selecting a response.

Materials & experimental systems

n/a	Involved in the study	
☒	☐	Antibodies
☒	☐	Eukaryotic cell lines
☒	☐	Palaeontology and archaeology
☒	☐	Animals and other organisms
☒	☐	Human research participants
☒	☐	Clinical data
☒	☐	Dual use research of concern

Methods

n/a	Involved in the study	
☒	☐	ChIP-seq
☒	☐	Flow cytometry
☒	☐	MRI-based neuroimaging
